# Deregulation between miR-29b/c and DNMT3A Is Associated with Epigenetic Silencing of the CDH1 Gene, Affecting Cell Migration and Invasion in Gastric Cancer

**DOI:** 10.1371/journal.pone.0123926

**Published:** 2015-04-15

**Authors:** He Cui, Ling Wang, Pihai Gong, Chengcheng Zhao, Shaodan Zhang, Kun Zhang, Rongping Zhou, Zhujiang Zhao, Hong Fan

**Affiliations:** 1 Department of Medical Genetics and Developmental Biology, Medical School of Southeast University, The Key Laboratory of Developmental Genes and Human Diseases, Ministry of Education, Southeast University, Nanjing, 210009, China; 2 The 3rd Affiliated Hospital of Harbin Medical University (Harbin Medical University Cancer Hospital), Harbin, 150081, China; 3 The Affiliated Jiangning Hospital with Nanjing Medical University, Nanjing, 210000, China; University of Alabama at Birmingham, UNITED STATES

## Abstract

The de-regulation of the miR-29 family and DNA methyltransferase 3A (DNMT3A) is associated with gastric cancer (GC). While increasing evidence indicates miR-29b/c could regulate DNA methylation by targeting DNMT3A, it is currently unknown if epigenetic silencing of miR-29b/c via promoter hypermethylation in GC is caused by abnormal expression of DNMT3A. Thus, we aimed to evaluate whether cross-talk regulation exists between miR-29b/c and DNMT3A and whether it is associated with a malignant phenotype in GC. First, wound healing and Transwell assays revealed that miR-29b/c suppresses tumor metastasis in GC. A luciferase reporter assay demonstrated that DNMT3A is a direct target of miR-29b/c. We used bisulfite genomic sequencing to analyze the DNA methylation status of miR-29b/c. The percentage of methylated CpGs was significantly decreased in DNMT3A-depleted cells compared to the controls. Furthermore, the involvement of DNMT3A in promoting GC cell migration was associated with the promoter methylation-mediated repression of CDH1. In 50 paired clinical GC tissue specimens, decreased miR-29b/c was significantly correlated with the degree of differentiation and invasion of the cells and was negatively correlated with DNMT3A expression. Together, our preliminary results suggest that the following process may be involved in GC tumorigenesis. miR-29b/c suppresses the downstream gene DNMT3A, and in turn, miR-29b/c is suppressed by DNMT3A in a DNA methylation-dependent manner. The de-regulation of both of miR-29b/c and DNMT3A leads to the epigenetic silencing of CDH1 and contributes to the metastasis phenotype in GC. This finding reveals that DNA methylation-associated silencing of miR-29b/c is critical for GC development and thus may be a therapeutic target.

## Introduction

Gastric cancer (GC) is the second most fatal malignancy worldwide. It accounts for a total of approximately 1 million new cases and 0.7 million deaths annually, over 70% of which occur in developing countries, particularly in East Asian countries [1]. Although curable if detected early, most GC patients are diagnosed with late stage disease. For patients with operable disease, conventional surgery and combination chemotherapies are indicated. However, the overall 5-year survival rate of GC patients is less than 30% [1, 2]. Notably, GC is often accompanied by peritoneal dissemination and metastasis to regional lymph nodes and distant organs through lymphatic and venous vessels [3]. Thus, identifying molecular aberrations in GC may improve our understanding of gastric carcinogenesis and help us subdivide patients into biologically and clinically relevant subgroups, as well as develop novel therapeutic strategies.

MicroRNAs (miRNAs) are a class of endogenous, small, non-coding regulatory RNAs of approximately 20–25 nucleotides that negatively regulate gene expression by inhibiting translation or inducing mRNA degradation through base pairing with the 3’ untranslated region (3’UTR) of target messenger RNAs (mRNAs) [4]. Altered expression levels of miRNAs have been reported in many cancers and result in aberrant expression of target genes that influence malignant behavior, such as proliferation, resistance to apoptosis and metastasis [5–7]. Increasing evidence shows that deregulated miRNAs (e.g., miR-17, miR-129, miR-148a, and miR-378) contribute to gastric carcinogenesis [8–10], which indicate that miRNAs could be used as diagnostic and prognostic biomarkers in GC.

The miR-29 family (miR-29s) is a conserved family of miRNAs that includes miR-29a/b/c. Decreased expression of miR-29s has been described in multiple cancers, including GC [11–14]. Previous studies demonstrate that miR-29s play a dominant role in GC cell proliferation, cell cycle progression, apoptosis, and cell motility [14, 15]. Potential targets of miR-29s contributing to the malignant GC phenotype include Cdc42, CCND2, and MMP2 [14, 15]. In addition, some studies have identified miR-29s as contributors to the regulation of DNA methylation by targeting DNMT3s in lung cancer [13]. Furthermore, several target genes, such as TCL-1, CDK6, laminin-1, and MCL-1, have also been reported in other cancer [16]. Notably, despite evidence demonstrating miRNA-29s can function as tumor-suppressor genes, one key question relating to miRNA-29s expression still remain partially unresolved. What are the mechanisms of control of miRNA-29s expression in GC cells? It has been reported that c-Myc is involved in miR-29a/b repression [17]. Identifying additional suppression mechanisms is of interest.

It is known that the transcriptional silencing of tumor suppressor genes (TSGs) by CpG island hypermethylation is a common hallmark of carcinogenesis. Interestingly, similar to protein-coding TSGs, a substantial number of miRNAs are regulated by promoter methylation [18–20]. Indeed, there has been an increasing number of studies showing that tumor suppressor miRNAs, such as miR-34b, miR-129, and miR-124, are frequently silenced by DNA methylation in GC [21–23]. Based on the CpG Island Searcher program analysis, our prediction showed that miRNA-29b/c contains CpG islands in their putative promoter regions. However, it is not yet clear if aberrant DNA hypermethylation accounts for the dysregulation of miR-29b/c. In addition, it is unknown whether a feedback regulation exists between miR-29b/c and DNMT3s. In fact, no previous study has examined the methylation status of miR-29b/c in GC cells, and none have explored the relationship between the methylation of miR-29b/c and levels of expression of DNMT3s. In this study, we found that miR-29b/c suppressed the expression of DNMT3A by targeting its 3’UTR, which contributed to inhibiting GC cell migration and invasion. On the other hand, DNMT3A down-regulated miR-29b/c via aberrant hypermethylation of the promoter. Thus, a potential feedback loop exists between miR-29b/c and DNMT3A, in which the down-regulation of miR-29b/c abolishes the suppression of DNMT3A. Meanwhile, the up-regulation of DNMT3A affects the expression of miR-29b/c by promoter methylation. These findings suggest a cross-talk between miR-29b/c and DNMT3A. Their imbalance and deregulation is cause by an epigenetic mechanism that may be involved in GC cell migration and invasion characteristics.

## Materials and Methods

### Cell culture

GC cell lines, including AGS and BGC-823, were obtained from the Cell Bank of the Chinese Academy of Science and maintained in RPMI-1640 medium supplemented with 10% fetal bovine serum (Invitrogen, Carlsbad, CA), 100 U/ml of penicillin and 100 mg/ml streptomycin (Invitrogen, Carlsbad, CA) in a humidified incubator with a 5% CO_2_ atmosphere at 37°C.

### Tissue samples

50 pairs of GC tissues and their adjacent non-cancerous tissue specimens were collected between 2011 and 2013 from the Jiangning Hospital of Nanjing. The clinical information of the patients with GC is shown in S1 Table. The study was approved by the Committee for Ethical Review of Research at the Jiangning Hospital of Nanjing in China, and the patients signed informed consent forms. All the tissue samples were obtained from patients with GC. They were collected during surgery and immediately snap frozen in liquid nitrogen until RNA and protein extraction.

### Reverse transcription reaction and quantitative real-time PCR (qPCR)

Total RNA was extracted from the cells and tissues harvested using Trizol reagent (Invitrogen, Carlsbad, CA) according to the manufacturer’s instruction.

To detect miRNA expression, a stem-loop RT-PCR was performed as previously described [24], and U6 small RNAs were used as an internal control. qPCR was carried out using SYBR Premix Ex Taq (Takara, Dalian, China) according to the manufacturer’s protocol. The relative expression was evaluated by the comparative CT method. The primer sequences of each gene are shown in S2 Table.

### Western blot

Western blots were performed using anti-DNMT3A (Abcam, Cambridge, UK), anti-CDH1 (Abcam, Cambridge, UK), anti-Vimentin (Santa Cruz Biotechnology, Santa Cruz, CA, USA) and anti-*β*-actin antibodies (Sigma, Ronkonkoma, NY, USA), and detection was performed with Super Signal chemiluminescence substrate (Pierce, Rockford, IL, USA).

### Transfection

miR-29b/c mimics/inhibitors and negative control molecules (scramble control mimic and inhibitor) were synthesized and purified by the GenePharma Company (Shanghai, China). The sequences are shown in S2 Table. They were transfected into the cells at a final concentration of 50 nM using Lipofectamine-2000 transfection reagent (Invitrogen, Carlsbad, USA) according to the manufacturer’s protocol. The medium was changed after 6 hours. The cells were cultured for 48 hours and harvested for analysis. The percent transfection efficiency was determined by an evaluation of the levels of miR-29b/c expression or knockdown following transfections (S1A and S1B Fig). The protocol for establishing the DNMT3A stable knockdown cells has been described in our previous work [25]. The expression of DNMT3A dramatically decreased in the BGC-shDNTM3A and AGS-shDNTM3A cells (S1C Fig) compared to the control cells.

### Wound healing, migration and invasion assays

Cell mobility was subjected to the wound healing assay analysis as described previously [26]. A scratch wound was generated using a 200 μl pipette tip on the confluent cell monolayers in six-well plates. The cells were then washed with fresh medium to remove floating cells, and the spread of the wound closure was observed after 48 hours and photographed under a microscope. The potential for migration and invasion of the transfected cells were evaluated by a Transwell assay. The cells were grown to 70% confluence and transfected for 24 hours with the miR-29b/c mimics or control mimics, and miR-29b/c inhibitor or control inhibitor. In the migration assay, the cells were cultured in 200 ml medium with 1% fetal bovine serum in the upper chamber of a non-coated transwell insert. In the lower chamber, 600 ml medium with 10% fetal bovine serum was used as a chemoattractant to encourage cell migration. In the invasion assay, the upper chamber of the transwell inserts were coated with 50 ml of 1.0 mg/ml Matrigel, and the cells were plated in the upper chamber of the Matrigel-coated transwell insert (Millipore, USA). After 24-hour incubation, the non-migrating or non-invading cells were gently removed with a cotton swab. All of the cells were stained using 0.1% crystal violet staining and counted in 5 fields under an inverted microscope. The independent experiments were repeated three times.

### Bisulfite genomic sequencing (BGS)

Genomic DNA was extracted from the cells using the phenol-chloroform method. Bisulfite treatment was performed by the CpGenome Universal DNA Modification Kit (Millipore, USA) following the manufacturer’s instructions. PCR products for bisulfite sequencing were gel-purified and subcloned into a pMD19-T vector system (Takara, Dalian, China). At least ten colonies were sequenced to assess the degree of methylation at each CpG site. The primers are listed in S2 Table.

### Luciferase reporter assay

The protocol for the luciferase reporter assay has been described previously [14]. The 3’UTR of human DNMT3A was PCR-amplified and cloned in between the Not I and Xba I sites of pGL-3 (Promega, USA). The BGC-823 cells were plated at a density of 5×10^5^ per well in a 12-well plate before the transfections. The cells were transfected with pGL-3 firefly luciferase reporters (1μg per well), pRL-TK (50 ng per well) and miR-29 mimics/inhibitor (50 nM) or negative control mimics/inhibitors (50 nM) using Lipofectamine-2000 transfection reagent (Invitrogen, Carlsbad, USA) according to the manufacturer’s protocol. Luciferase activity was tested 48 hours after the transfection using the Dual-Luciferase Activity Assay System (Promega, USA). All the experiments were performed as three independent replicates.

### Statistical analysis

The independent Student’s *t*-test was used to compare the results, which are expressed as the mean±s.d. between any two preselected groups. To determine correlations between the variables, *Pearson*’s correlation coefficient was calculated. A *P*-value less than 0.05 was considered statistically significant.

## Results

### miR-29b/c is required and sufficient for GC cell migration

**Fig 1:**
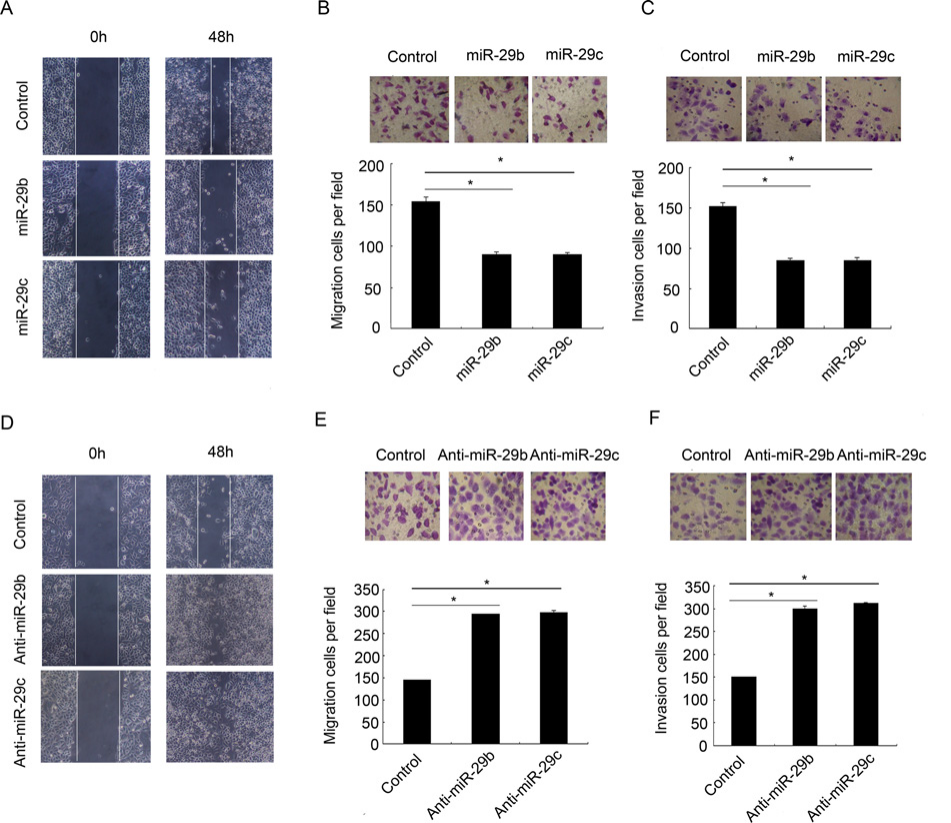
The effect of miR-29b/c on GC cell invasion and migration. (A) Wound healing assays of the confluent layers of negative control mimics or miR-29b/c mimics-transfected BGC-823 cells. Images were acquired at 0 and 48 hours after wounding. (B and C) Representative images (upper) and bar graphs (bottom) depicting the migration (B) and invasion (C) ability of BGC-823 cells after the 48-hour transfection of negative control mimics or miR-29b/c mimics (*P<0.05). (D) Wound healing assays on the confluent layers of negative control inhibitors or miR-29b/c inhibitors-transfected BGC-823 cells. Images were acquired at 0 and 48 hours after wounding. (E and F) Representative images (upper) and bar graphs (bottom) depicting the migration (E) and invasion (F) ability of BGC-823 cells after the 48-hour transfection of negative control inhibitors or miR-29b/c inhibitors (*P<0.05). Number of cells that migrated or invaded was counted in five fields. The migration and invasion rate are represented as cell number per field.

Previous studies imply that miR-29b/c is critical for GC cell invasion. To further evaluate whether miR-29b/c is responsible for GC cell migration, an *in vitro* scratch wound healing assay and Transwell assay were performed. After transiently transfecting the miR-29b/c mimics or negative control into the BGC-823 cells, the wound healing assay showed that the cells with the forced expression of miR-29b/c displayed a notably slower recovery compared with the control cells (Fig 1A). Similarly, the Transwell migration assay showed that the overexpression of miR-29b/c was associated with significantly less migration than the controls (*P*<0.05, Fig 1B). Moreover, consistent with other data in HGC-207 and MGC-803 GC cells [14], the miR-29b/c overexpression cells also revealed a significant reduction in invasive ability in the Matrigel invasion assay (*P*<0.05, Fig 1C). These results suggest that miR-29b/c is not only important for GC cell invasion but also for cell migration. To further confirm the suppressive effects of miR-29b/c on GC cell migration and invasion, the BGC-823 cells were transiently transfected with the miR-29b/c inhibitors or a negative control. The deletion of miR-29b/c significantly increased the migratory and invasive capabilities of the GC cells, which was assessed by wound healing (Fig 1D) and a Transwell assay (*P*<0.05, Fig 1E and Fig 1F). Collectively, these results indicate that miR-29b/c effectively abolished GC cell migration and invasion, which therefore contributed to the early stages of the malignant progression of GC.

### DNMT3A is a direct transcriptional target of miR-29b/c in GC

**Fig 2:**
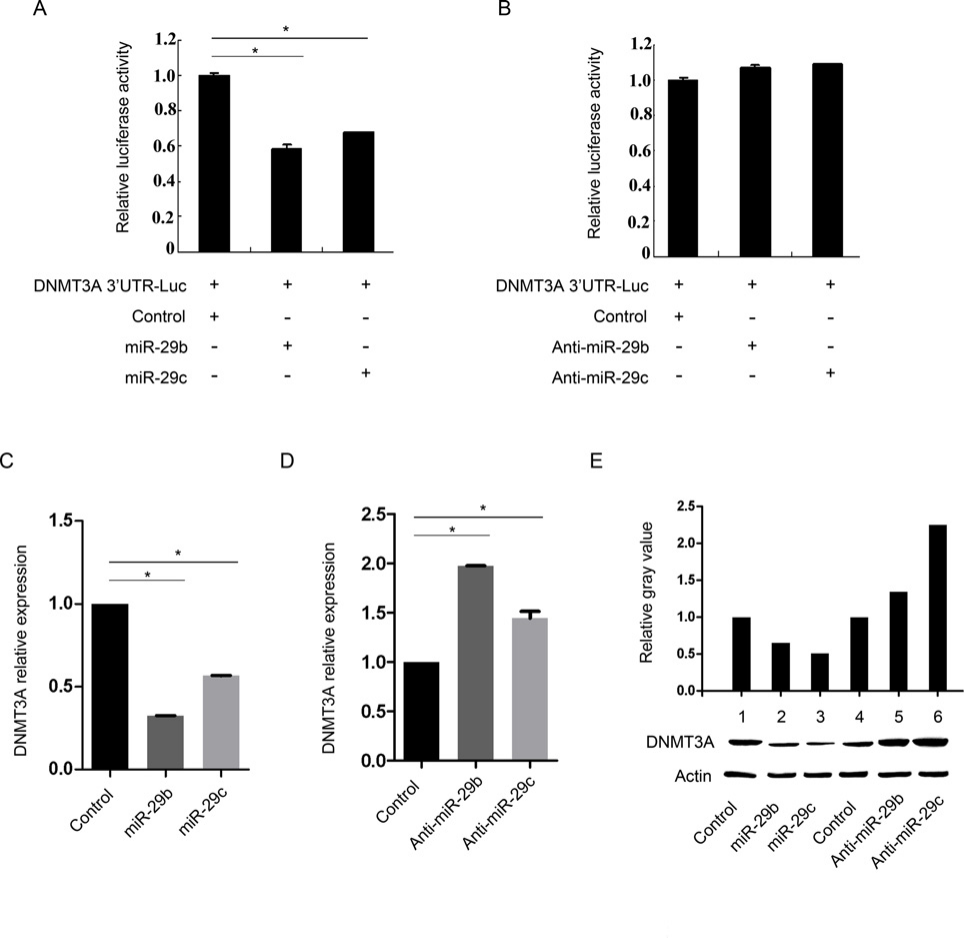
miR-29b/c directly targets DNMT3A. (A and B) Effects of miR-29b/c on the transcriptional activity of DNMT3A detected by luciferase assay. The 3’UTR of DNMT3A was cloned into the pGL3-basic luciferase reporter vector (DNMT3A 3’UTR-Luc), which was co-transfected with miR-29b/c mimics or negative control mimics (A), or miR-29b/c inhibitors or negative control inhibitors (B) into BGC-823 cells and assayed for luciferase activity. Firefly luciferase values were normalized to Renilla luciferase activity derived from pRL-TK and plotted as relative luciferase activity (*P<0.05). The results are expressed as the mean±s.d. of at least three independent experiments. (C and D) qRT-PCR analysis of the DNMT3A relative expression in BGC-823 cells transfected with miR-29b/c mimics (C)/inhibitors (D) or negative control oligonucleotide (*P<0.05). (E) Western blot analysis of DNMT3A expression in BGC-823 cells transfected with miR-29b/c mimics (lane 1, 2, 3) /inhibitors (lane 4, 5, 6) or negative control oligonucleotide. *β*-actin was used as a loading control. The band intensities were quantified and normalized to *β*-actin intensities with ImageJ software.

To understand the mechanism underlying the effect of miR-29b/c on cell migration and invasion, we investigated the targets of miR-29b/c. DNMT3A 3’UTR is complementary to miR-29b/c and is a direct target gene of miR-29b/c, thus we carried out a reporter assay in BGC-823 cells. The DNMT3A mRNA 3’UTR was inserted into the downstream region of a luciferase reporter gene from the pGL-3 vector (namely DNMT3A 3’UTR-Luc). The constructs were then cotransfected with pRL-TK and the miR-29b/c mimics or the negative control mimics into the BGC-823 cells. As shown in Fig 2A, the relative luciferase activity was significantly reduced in the pGL-3 vectors with the DNMT3A 3’UTR (*P*<0.05). However, the constructs cotransfected with the miR-29b/c inhibitor did not affect the luciferase activity of the pGL-3 vectors with the DNMT3A 3’UTR compared to the constructs cotransfected with the negative control inhibitor (Fig 2B). To further validate this miRNA-target interaction, quantitative RT-PCR (qRT-PCR) and western blots were performed to confirm the regulation of DNMT3A by miR-29b/c. We transiently transfected the miR-29b/c mimics or negative control mimics into the BGC-823 cells. The increased miR-29b/c reduced the DNMT3A expression at the mRNA (Fig 2C) and protein levels (Fig 2E, lane1-3). Conversely, the miR-29b/c down-regulation by its inhibitor increased the DNMT3A expression at the mRNA (Fig 2D) and protein levels (Fig 2E, lane4-6). Collectively, these findings indicate that DNMT3A is a direct transcriptional target of miR-29b/c and is negatively associated with miR-29b/c expression in GC cells.

### miR-29b/c is suppressed by DNMT3A in a DNA methylation-dependent manner

**Fig 3:**
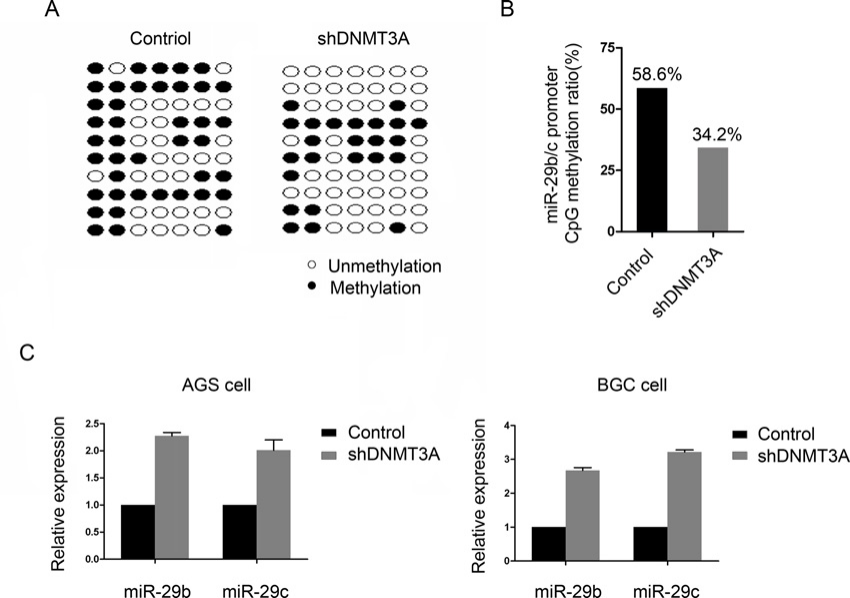
DNMT3A suppresses miR-29b/c in a DNA methylation-dependent manner. (A and B) Mapping of the methylation status of individual CpG sites in the miR-29b/c promoter by BGS assay in BGC-control and BGC-shDNTM3A cells. The regions spanning the CpG island with 7 CpG sites were analyzed. Black circles represent the methylated CpG site; white circles represent the unmethylated CpG site. Each row represents bisulfite sequencing of miR-29b/c promoter for a single analyzed random clone. (C) qRT-PCR analysis of miR-29b/c expression in both AGS and BGC-823 DNMT3A-knockdown cells.

Given that aberrant hypermethylation is critical for miRNA down-regulation, we speculated that negative feedback exists between miR-29b/c and DNMT3A. To test this hypothesis, the presence of CpG island methylation in the miR-29b/c promoter region was evaluated by a BGS analysis in the DNMT3A-knockdown cells. As shown in Fig 3A, 7 individual CpG sites within the CpG island regions (5 kb upstream of miR-29b/c) were sequenced to identify methylated cytosine residues. The frequency of miR-29b/c promoter methylation in DNMT3A-knockdown cells was 34.2%, which was significantly lower than the control cells (58.6%; Fig 3B). This result indicates that the transcriptional silencing of miR-29b/c in GC cells is associated with the presence of CpG island hypermethylation, which was confirmed by measuring mature miR-29b/c by qRT-PCR. There was significantly increased miR-29b/c expression in the AGS-shDNMT3A and BGC-shDNMT3A cells compared with their controls (Fig 3C). These findings indicate an important molecular basis for miR29-b/c down-regulation and support the hypothesis that there is cross-talk between miR-29b/c and DNMT3A.

### DNMT3A promotes GC cell migration

**Fig 4:**
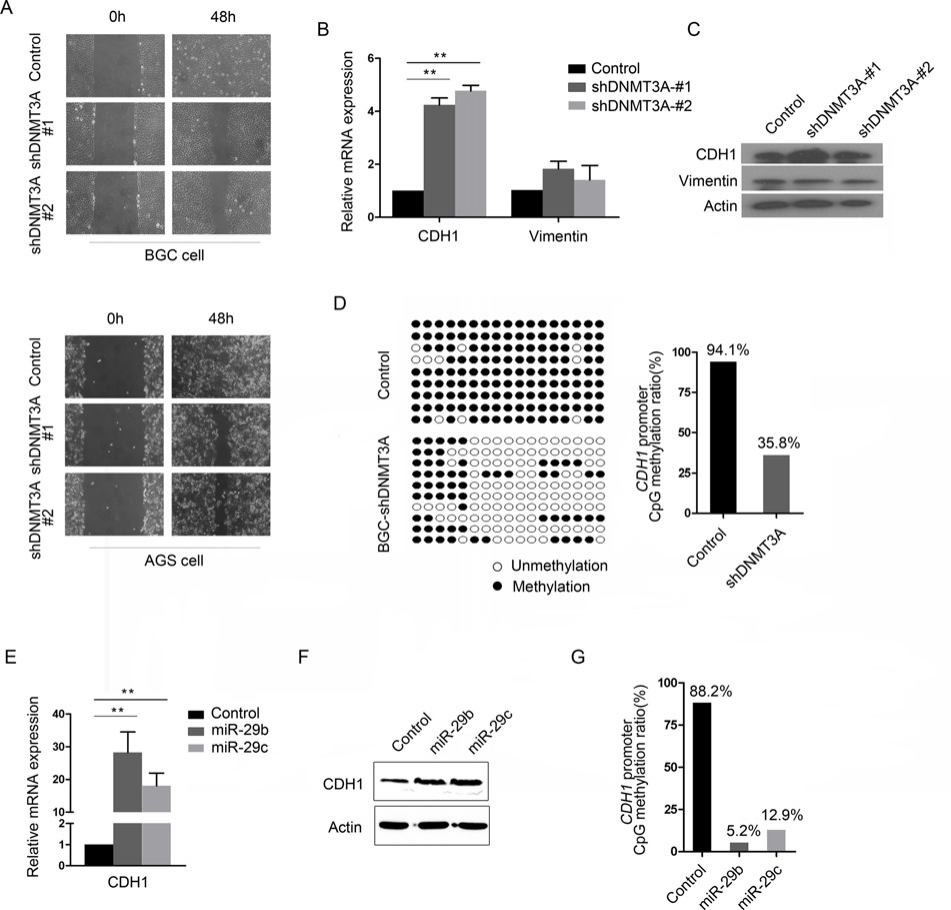
Both of DNMT3A and miR-29b/c are involved in GC migration. (A) Cell migratioin rates of DNTM3A knockdown BGC or AGS cells were compared with control via wound healing assays. Microscopic observation was recorded at 0 and 48 hours after scratching the surface of a confluent layer of cells. (B and C) qRT-PCR (B) and western blot (C) analysis of CDH1 or Vimentin expression in DNMT3A-knockdown BGC-823 cells. *β*-actin was used as a loading control. (D) Mapping of the methylation status of individual CpG sites in the CDH1 promoter by BGS assay (Left) and bar graphs depicting the CDH1 promoter methylation rates in BGC-control and BGC-shDNTM3A cells (right). The regions spanning the CpG island with 17 CpG sites were analyzed. Black circles represent the methylated CpG sites; white circles represent the unmethylated CpG sites. Each row represents bisulfite sequencing of CDH1 promoter for a single analyzed random clone. (E and F) qRT-PCR (E) and western blot (F) analysis of CDH1 or Vimentin expresion in BGC-823 cells after miR-29b/c mimics or negative control mimics 48-hour transfection(**P<0.01). *β*-actin was used as a loading control. (G) Bar graphs depicting the CDH1 promoter methylation rates in BGC-823 cells after miR-29b/c mimics or negative control mimics 48-hour transfection. The regions spanning the CpG island with 17 CpG sites were analyzed.

The molecular connection between miR-29b/c and DNMT3A raised the question of DNMT3A involvement in GC cell migration. We further applied *in vitro* assays to determine the functional changes in cell behavior following altered expression of DNMT3A. The wound healing assay demonstrated a notably slower recovery in the BGC-shDNMT3A cells compared with the control cells (Fig 4A, Top), but only a modest recovery in the AGS-shDNMT3A cells compared with the control cells (Fig 4A, bottom). These results indicate that DNMT3A is important for cell mobility. Given that cell adhesion molecules are important for cell motility, the expression of CDH1 and Vimentin were examined by qRT-PCR and western blot. Knockdown of DNMT3A expression significantly increased the CDH1 expression at both the mRNA and protein levels, but did not have a remarkable effect on the expression of Vimentin (Fig 4B and 4C), suggesting that CDH1 may be a target of DNMT3A-mediated dysregulation of cell motility. Furthermore, we carried out a BGS assay on the CDH1 gene in the DNMT3A-knockdown cells. As shown in Fig 4D, the percentage of methylated CpGs located within CDH1 was lower in the DNMT3A-depleted cells than in the control cells (35.8% vs. 94.1%). These results indicate that the abnormal expression of DNMT3A leads to an epigenetic silencing of CDH1.

### miR-29b/c is involved in the suppression of CDH1

To further investigate the effect of altered miR-29b/c expression on CDH1, we transiently transfected the miR-29b/c mimics or negative control mimic into BGC-823 cells. Compared with the controls, the forced expression of miR-29b/c significantly increased the expression of CDH1 at both the mRNA and protein levels (Fig 4E and 4F). Subsequently, the methylation status of the promoter region of CDH1 was examined using a BGS assay in the miR-29b/c mimics or negative control mimic transiently transfected BGC-823 cells. The results showed that the frequency of CDH1 promoter methylation in the miR-29b/c overexpression cells was 5.2% or 12.9%, which was significantly lower than the level measured in the control cells (88.2%; Fig 4G). These data were consistent with the result that DNMT3A knockdown mediates the up-regulation of CDH1 and demonstrates that the de-regulation of miR-29b/c and DNMT3A are involved in GC cell migration and invasion.

### Decreased expression of miR-29b and miR-29c in GC tissues

**Fig 5:**
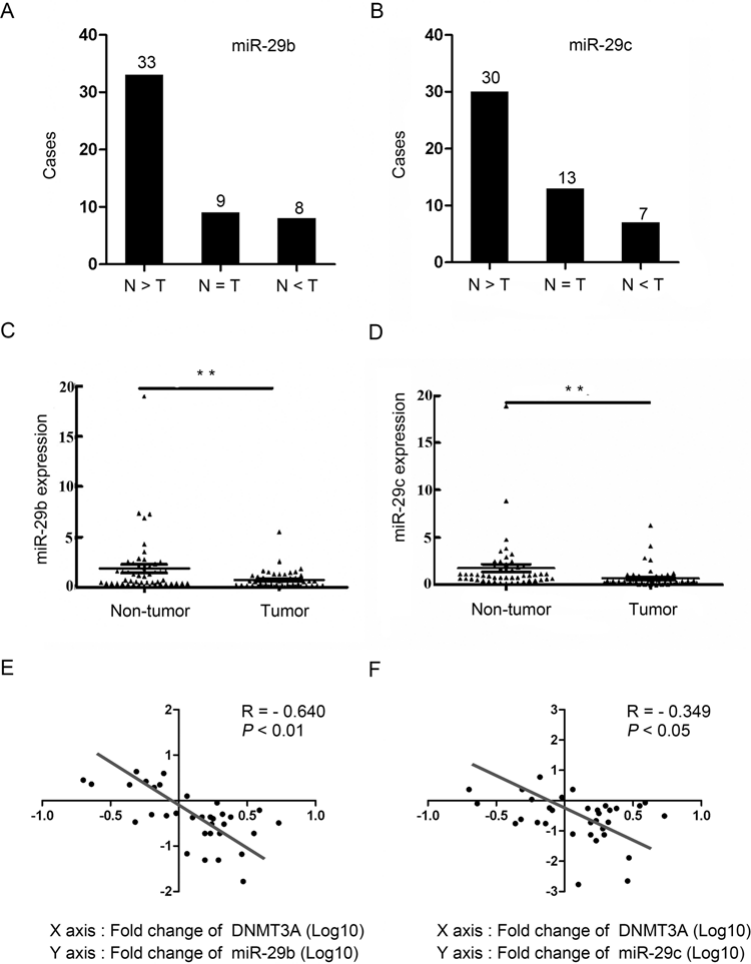
Decreased miR-29b/c in GC tissue samples. (A and B) qRT-PCR analysis showing miR-29b (A) or miR-29c (B) in 50 GC tissues (T) and paired non-tumor tissues (N). Clinical samples were divided into three groups based on miRNA relative expression scores greater or less than two fold: N>T, N = T and N<T. The case number is shown in every group. (C and D) Scatter plots of miR-29b (C) or miR-29c (D) fold changes in GC and their paired non-tumor tissues. In both panels, the horizontal lines represent the median and the vertical bars representing the range of the data (**P<0.01). (E and F) Correlation between DNMT3A and miR-29b (E) or miR-29c (F) expression in 33 clinical samples, with linear regression lines and Pearson correlation significance (miR-29b: R = -0.640; **P<0.01; miR-29c: R = -0.349; *P<0.05; Pearson *χ2* test).

The expression of miR-29b/c in 50 GC tumors and paired non-tumor tissues was examined by qRT-PCR. Compared to the paired non-tumor tissues, the frequency of miR-29b and miR-29c down-regulation (defined as a greater than two-fold decrease) was 66% (33/50) and 60% (30/50), respectively (Fig 5A and 5B). The average fold change of miR-29b and miR-29c was significantly lower in the tumor tissues than in the non-tumor tissues (*P*<0.01, Fig 5C and 5D). To explore the clinicopathological significance of the miR-29b and miR-29c expression patterns in GC tumorigenesis, the clinical features of GC patients were analyzed. All the 43 patients analyzed were categorized into two groups according to miR-29b and miR-29c expression levels. Decreased miR-29b strongly correlated with the degree of tumor cell differentiation and invasion, whereas decreased miR-29c only correlated with the tumor invasion (Table 1). Furthermore, the relationship between miR-29b/c expression and DNMT3A expression was tested in 33 GC cases. An association study showed that DNMT3A expression was negatively correlated with miR-29b (*R* = -0.640, *P*<0.01, Fig 5E) and miR-29c (*R* = -0.349, *P*<0.05, Fig 5F). Collectively, these data indicate that miR-29b/c might play an important role in the progression of gastric carcinogenesis.

**Table 1:** Clinicopathological correlation of miR-29b/c expression in 43 GC cases.

Feature	miR-29b			miR-29c		
	N>T	N≤T	*P*-value	N>T	N≤T	*P*-value
**Gender**						
Female	7	5		8	4	
Male	25	6	0.133	25	6	0.330
**Histologic grade**						
Poor	23	2		19	3	
Moderate	9	9		14	7	
High ^a^	0	0	**0.004** *	0	0	0.162
**Invasion degree**						
Early stage	2	4		2	4	
Progression	30	7	**0.029** *	31	6	**0.020** *
**Lymph node metastasis**						
Yes	27	6		28	6	
No	6	4	0.206	5	4	0.177

* Significant differences are shown in bold. N: non-tumor tissues; T: tumor tissues.

^**a**^ No cases in this pathological classification

## Discussion

The conserved miR-29 family, including miR-29a, miR-29b and miR-29c, is implicated in several cellar processes, such as proliferation, differentiation, apoptosis and metastasis, making them a well-analyzed family of miRNAs in tumorigenesis [16]. Previous studies have shown that increased expression of miR-29a is associated with better overall survival rates in stage II colon cancer patients [27] and lower levels of miR-29b could promote prostate cancer metastasis by regulating epithelial-mesenchymal transition signaling pathways [28]. In addition, miR-29c functions as a metastasis suppressor that inhibits lung cancer cell adhesion to the extracellular matrix and migration *in vitro* and *in vivo* [29]. In GC, significantly reduced levels of miR-29b and miR-29c, in particular, have been observed compared to miR-29a [14], suggesting that miR-29b/c may play a more important role. Thus, miR-29b/c was selected for analysis in this study. In the present study, we showed an increased miR-29b/c suppresses the migration and invasion of BGC-823 cells using a wound healing assay and a Transwell assay. These results are consistent with other reported data obtained from SGC-7901, HGC-27 and MGC-803 GC cells [14, 15]. Given that miR-29b/c also play roles in proliferation and apoptosis in GC, we assessed the ability of cell growth and the levels of cell apoptosis in BGC-823 cells. The results showed that there is no difference in proliferation at 48 hours for miR-29b/c mimics or inhibitors-transfected cells, compared with the negative control cells (*P*>0.05, S2A and S2B Fig). Furthermore, Annexin-V staining demonstrated no dramatic increase in the levels of apoptosis in the miR-29b/c mimics-transfected cells after 48 hours of incubation (S2C Fig). In addition, the cell cycle analysis showed no significant differences in G1, S, G2/M phases after treatment with the miR-29b/c mimics or negative control mimics for 48 hours (*P*>0.05, S2D Fig). These data suggest that miR-29b/c slows wound area recovery at 48 hours mainly because of the decreased cell motility abilities.

miRNAs exert their functions mainly by targeting the 3’UTRs of different genes. However, the detailed molecular mechanisms of miR-29b/c related to malignant GC development are poorly understood. Notably, miR-29b/c shares the same complementarity to sites in the 3’UTR of DNMT3A, which was predicted by target prediction programs including TargetScan, Miranda and miRBase. It is not yet known whether miR-29b/c regulates the abnormal methylation of genes associated with metastasis by interacting with DNMT3A during the development of GC. Therefore, we performed a luciferase reporter assay and found that a high DNMT3A expression was associated with low miR-29b/c expression in GC cells, indicating DNMT3A is a direct transcriptional target of miR-29b/c. However, the molecular basis that leads to the imbalance of miR-29b/c in GC remains unknown. miR-29 proximal promoters have binding sites for several transcription factors, such as c-Myc, and CEBPA, which contribute to the deregulation of miR-29s [30, 31]. However, research on the epigenetic regulation of miRNA-29s has not been reported.

In eukaryotic cells, there are three enzymatically active DNA methyltransferases (DNMTs), DNMT1, DNMT3A and DNMT3B. The expression of DNMT3A in GC is significantly higher than that of DNMT3B [32, 33]. Moreover, only increased DNMT3A expression is significantly associated with a shorter disease-free survival period in GC [34], which indicates DNMT3A plays more important roles in gastric carcinogenesis. Thus, we aimed to investigate whether negative-feedback regulations exist between miR-29b/c and DNMT3A. In this study, we used a BGS assay to analyze the DNA methylation status of miR-29b/c in DNMT3A RNAi GC cells. Indeed, the decreased miR-29b/c was partially due to DNMT3A-mediated hypermethylation. Furthermore, we confirmed that DNMT3A is involved in promoting GC cell migration via down-regulating CDH1. Meanwhile, we also showed that miR-29b/c is involved in the suppression of CDH1. A hallmark of cancer metastasis is the loss of CDH1 expression [35], and hypermethylation within the CDH1 promoter region is also observed in GC [36]. Thus, our results demonstrate that both miR-29b/c and DNMT3A are associated with CDH1 down-regulation through DNMT3A-mediated promoter hypermethylation. The role of other DMNTs, for example DNMT3B, in regulating miR-29b/c needs to be examined in future studies.

In GC specimens, we examined the expression pattern of miR-29b/c in 50 pairs of GC tissues. Decreased miR-29b/c (fold-change cutoff: 2.0) was significantly correlated with the differentiation and invasion degree in GC, which suggests that miR-29b/c plays a critical role in GC malignant maintenance and directly demonstrates the clinical significance of miR-29b/c in GC progression. In summary, our study reveals that there may be cross-talk between miR-29b/c and DNMT3A. An imbalance of these factors may be involved in GC migration and invasion phenotypes. This imbalance may include low-level expression of miR-29b/c could abolish suppression of DNMT3A and high levels of DNMT3A further affects the expression of miR-29b/c by an epigenetic mechanism. These findings may be beneficial for the development of new treatment options for GC that target miR-29b/c and its downstream gene DNMT3A.

## Supporting Information

S1 Fig The percentage transfection efficiency was provided by evaluation of levels of miR-29b/c or DNMT3A. (A and B) qRT-PCR was performed to detect the relative expression of miR-29b/c in BGC-823 cells with mimics (A) or inhibitors (B) after 48-hour transfection. (C) RT-PCR and western blots were performed to detect the efficiency of DNMT3A knockdown in BGC and AGS cells. *β*-actin was used as a loading control.
(TIF)

S2 Fig The Role of miR-29b/c in the BGC-823 cells proliferation, apoptosis and cell cycle. (A) The cell growth rates of miR-29b/c overexpression were detected by CCK-8 proliferation assay. miR-29b/c overexpression showed no remarkable difference at 48 hours compared to negative control cells (*P*>0.05). (B) The cell growth rates of miR-29b/c inhibition were detected by CCK-8 proliferation assay. The suppression of miR-29b/c showed no significant changes at 48 hours compared to negative control cells (*P*>0.05). (C) Apoptosis assay showing no dramatic induction of apoptosis by miR-29b/c overexpression at 48 hours compared to negative control cells. The biparametric histogram shows cell in early (bottom right quadrant) and the late apoptotic states (upper right quadrant). (D) The cell cycle assay was performed by flow cytometry on BGC-823 cells after miR-29b/c mimics or negative control mimics treatment for 48 hours. The percentages of miR-29b/c overexpression or control cells in the G1, S, and G2/M are shown in the bar chart as the mean±s.d. of three independent experiments. Compared with control cells, there was no significant effect on the cell cycle after miR-29b/c overexpression.
(TIF)

S1 Method Cell growth and apoptosis assay. (DOC)

S2 Method Cell cycle assay. (DOC)

S1 Table Clinical features of patients with gastric cancer. (DOC)

S2 Table miRNA mimic and inhibitor sequences and primers used in this study. (DOC)

## References

[1] Jemal A, Bray F, Center MM, Ferlay J, Ward E, Forman D. Global cancer statistics. CA Cancer J Clin. 2011;61: 69–90. 10.3322/caac.20107 21296855

[2] Lordick F, Siewert JR. Recent advances in multimodal treatment for gastric cancer: a review. Gastric Cancer. 2005;8: 78–85. 15864714 10.1007/s10120-005-0321-z

[3] Maehara Y, Moriguchi S, Kakeji Y, Kohnoe S, Korenaga D, Haraguchi M, et al Pertinent risk factors and gastric carcinoma with synchronous peritoneal dissemination or liver metastasis. Surgery. 1991;110: 820–823. 1948650

[4] Bartel DP. MicroRNAs: target recognition and regulatory functions. Cell. 2009;136: 215–233. 10.1016/j.cell.2009.01.002 19167326 PMC3794896

[5] Zhang H, Li Y, Lai M. The microRNA network and tumor metastasis. Oncogene. 2010;29: 937–948. 10.1038/onc.2009.406 19935707

[6] Cho WC. OncomiRs: the discovery and progress of microRNAs in cancers. Mol Cancer. 2007;6: 60 17894887 10.1186/1476-4598-6-60 PMC2098778

[7] Cheng AM, Byrom MW, Shelton J, Ford LP. Antisense inhibition of human miRNAs and indications for an involvement of miRNA in cell growth and apoptosis. Nucleic acids research. 2005;33: 1290–1297. 15741182 10.1093/nar/gki200 PMC552951

[8] Xia J, Guo X, Yan J, Deng K. The role of miR-148a in gastric cancer. J Cancer Res Clin Oncol. 2014;140: 1451–1456. 24659367 10.1007/s00432-014-1649-8 PMC11823805

[9] Fesler A, Zhai H, Ju J. miR-129 as a novel therapeutic target and biomarker in gastrointestinal cancer. Onco Targets Ther. 2014;7: 1481–1485. 10.2147/OTT.S65548 25187728 PMC4149397

[10] Wang JL, Hu Y, Kong X, Wang ZH, Chen HY, Xu J, et al Candidate microRNA biomarkers in human gastric cancer: a systematic review and validation study. PloS one. 2013;8: e73683 10.1371/journal.pone.0073683 24040025 PMC3767766

[11] Garzon R, Heaphy CE, Havelange V, Fabbri M, Volinia S, Tsao T, et al MicroRNA 29b functions in acute myeloid leukemia. Blood. 2009;114: 5331–5341. 10.1182/blood-2009-03-211938 19850741 PMC2796138

[12] Sengupta S, den Boon JA, Chen IH, Newton MA, Stanhope SA, Cheng YJ, et al MicroRNA 29c is down-regulated in nasopharyngeal carcinomas, up-regulating mRNAs encoding extracellular matrix proteins. Proceedings of the National Academy of Sciences. 2008;105: 5874–5878. 10.1073/pnas.0801130105 18390668 PMC2311339

[13] Fabbri M, Garzon R, Cimmino A, Liu Z, Zanesi N, Callegari E, et al MicroRNA-29 family reverts aberrant methylation in lung cancer by targeting DNA methyltransferases 3A and 3B. Proceedings of the National Academy of Sciences. 2007;104: 15805–15810. 17890317 10.1073/pnas.0707628104 PMC2000384

[14] Gong J, Li J, Wang Y, Liu C, Jia H, Jiang C, et al Characterization of microRNA-29 family expression and investigation of their mechanistic roles in gastric cancer. Carcinogenesis. 2014;35: 497–506. 10.1093/carcin/bgt337 24130168

[15] Lang N, Liu M, Tang QL, Chen X, Liu Z, Bi F. Effects of microRNA-29 family members on proliferation and invasion of gastric cancer cell lines. Chin J Cancer. 2010;29: 603–610. 20507733 10.5732/cjc.009.10597

[16] Wang Y, Zhang X, Li H, Yu J, Ren X. The role of miRNA-29 family in cancer. Eur J Cell Biol. 2013;92: 123–128. 10.1016/j.ejcb.2012.11.004 23357522

[17] Mott JL, Kurita S, Cazanave SC, Bronk SF, Werneburg NW, Fernandez-Zapico ME. Transcriptional suppression of mir-29b-1/mir-29a promoter by c-Myc, hedgehog, and NF-kappaB. J Cell Biochem. 2010;110: 1155–1164. 10.1002/jcb.22630 20564213 PMC2922950

[18] Han L, Witmer PD, Casey E, Valle D, Sukumar S. DNA methylation regulates MicroRNA expression. Cancer Biol Ther. 2007;6: 1284–1288. 17660710 10.4161/cbt.6.8.4486

[19] Lujambio A, Esteller M. CpG island hypermethylation of tumor suppressor microRNAs in human cancer. Cell Cycle. 2007;6: 1455–1459. 17581274

[20] Lujambio A, Ropero S, Ballestar E, Fraga MF, Cerrato C, Setien F, et al Genetic unmasking of an epigenetically silenced microRNA in human cancer cells. Cancer Res. 2007;67: 1424–1429. 17308079 10.1158/0008-5472.CAN-06-4218

[21] Ueda T, Volinia S, Okumura H, Shimizu M, Taccioli C, Rossi S, et al Relation between microRNA expression and progression and prognosis of gastric cancer: a microRNA expression analysis. Lancet Oncol. 2010;11: 136–146. 10.1016/S1470-2045(09)70343-2 20022810 PMC4299826

[22] Tsai KW, Wu CW, Hu LY, Li SC, Liao YL, Lai CH, et al Epigenetic regulation of miR-34b and miR-129 expression in gastric cancer. Int J Cancer. 2011;129: 2600–2610. 10.1002/ijc.25919 21960261

[23] Ando T, Yoshida T, Enomoto S, Asada K, Tatematsu M, Ichinose M, et al DNA methylation of microRNA genes in gastric mucosae of gastric cancer patients: its possible involvement in the formation of epigenetic field defect. Int J Cancer. 2009;124: 2367–2374. 10.1002/ijc.24219 19165869

[24] Chen C, Ridzon DA, Broomer AJ, Zhou Z, Lee DH, Nguyen JT, et al Real-time quantification of microRNAs by stem-loop RT-PCR. Nucleic Acids Res. 2005;33: e179 16314309 10.1093/nar/gni178 PMC1292995

[25] Zhao Z, Wu Q, Cheng J, Qiu X, Zhang J, Fan H. Depletion of DNMT3A suppressed cell proliferation and restored PTEN in hepatocellular carcinoma cell. J Biomed Biotechnol. 2010;2010: 737535 10.1155/2010/737535 20467490 PMC2868982

[26] Rodriguez LG, Wu X, Guan JL. Wound-healing assay. Methods Mol Biol. 2005;294: 23–29. 15576902 10.1385/1-59259-860-9:023

[27] Weissmann-Brenner A, Kushnir M, Lithwick Yanai G, Aharonov R, Gibori H, Purim O, et al Tumor microRNA-29a expression and the risk of recurrence in stage II colon cancer. Int J Oncol. 2012;40: 2097–2103. 10.3892/ijo.2012.1403 22426940

[28] Ru P, Steele R, Newhall P, Phillips NJ, Toth K, Ray RB. miRNA-29b suppresses prostate cancer metastasis by regulating epithelial-mesenchymal transition signaling. Mol Cancer Ther. 2012;11: 1166–1173. 10.1158/1535-7163.MCT-12-0100 22402125

[29] Wang H, Zhu Y, Zhao M, Wu C, Zhang P, Tang L, et al miRNA-29c suppresses lung cancer cell adhesion to extracellular matrix and metastasis by targeting integrin beta1 and matrix metalloproteinase2 (MMP2). PLoS One. 2013;8: e70192 10.1371/journal.pone.0070192 23936390 PMC3735565

[30] Chang TC, Yu D, Lee YS, Wentzel EA, Arking DE, West KM, et al Widespread microRNA repression by Myc contributes to tumorigenesis. Nat Genet. 2008;40: 43–50. 18066065 10.1038/ng.2007.30 PMC2628762

[31] Eyholzer M, Schmid S, Wilkens L, Mueller BU, Pabst T. The tumour-suppressive miR-29a/b1 cluster is regulated by CEBPA and blocked in human AML. Br J Cancer. 2010;103: 275–284. 10.1038/sj.bjc.6605751 20628397 PMC2906742

[32] Yang J, Wei X, Wu Q, Xu Z, Gu D, Jin Y, et al Clinical significance of the expression of DNA methyltransferase proteins in gastric cancer. Mol Med Rep. 2011;4: 1139–1143. 10.3892/mmr.2011.578 21887466

[33] Ding WJ, Fang JY, Chen XY, Peng YS. The expression and clinical significance of DNA methyltransferase proteins in human gastric cancer. Dig Dis Sci. 2008;53: 2083–2089. 10.1007/s10620-007-0145-2 18253830

[34] Cao XY, Ma HX, Shang YH, Jin MS, Kong F, Jia ZF, et al DNA methyltransferase3a expression is an independent poor prognostic indicator in gastric cancer. World J Gastroenterol. 2014;20: 8201–8208. 10.3748/wjg.v20.i25.8201 25009393 PMC4081693

[35] Thiery JP, Acloque H, Huang RY, Nieto MA. Epithelial-mesenchymal transitions in development and disease. Cell. 2009;139: 871–890. 10.1016/j.cell.2009.11.007 19945376

[36] Tamura G, Yin J, Wang S, Fleisher AS, Zou T, Abraham JM, et al E-Cadherin gene promoter hypermethylation in primary human gastric carcinomas. J Natl Cancer Inst. 2000;92: 569–573. 10749913 10.1093/jnci/92.7.569

