# Supplementary figures and images for: Deregulation between miR-29b/c and DNMT3A Is Associated with Epigenetic Silencing of the CDH1 Gene, Affecting Cell Migration and Invasion in Gastric Cancer
Source: PLoS One. 2015 Apr 15;10(4):e0123926. doi: 10.1371/journal.pone.0123926 (PMC4398372; doi:10.1371/journal.pone.0123926)

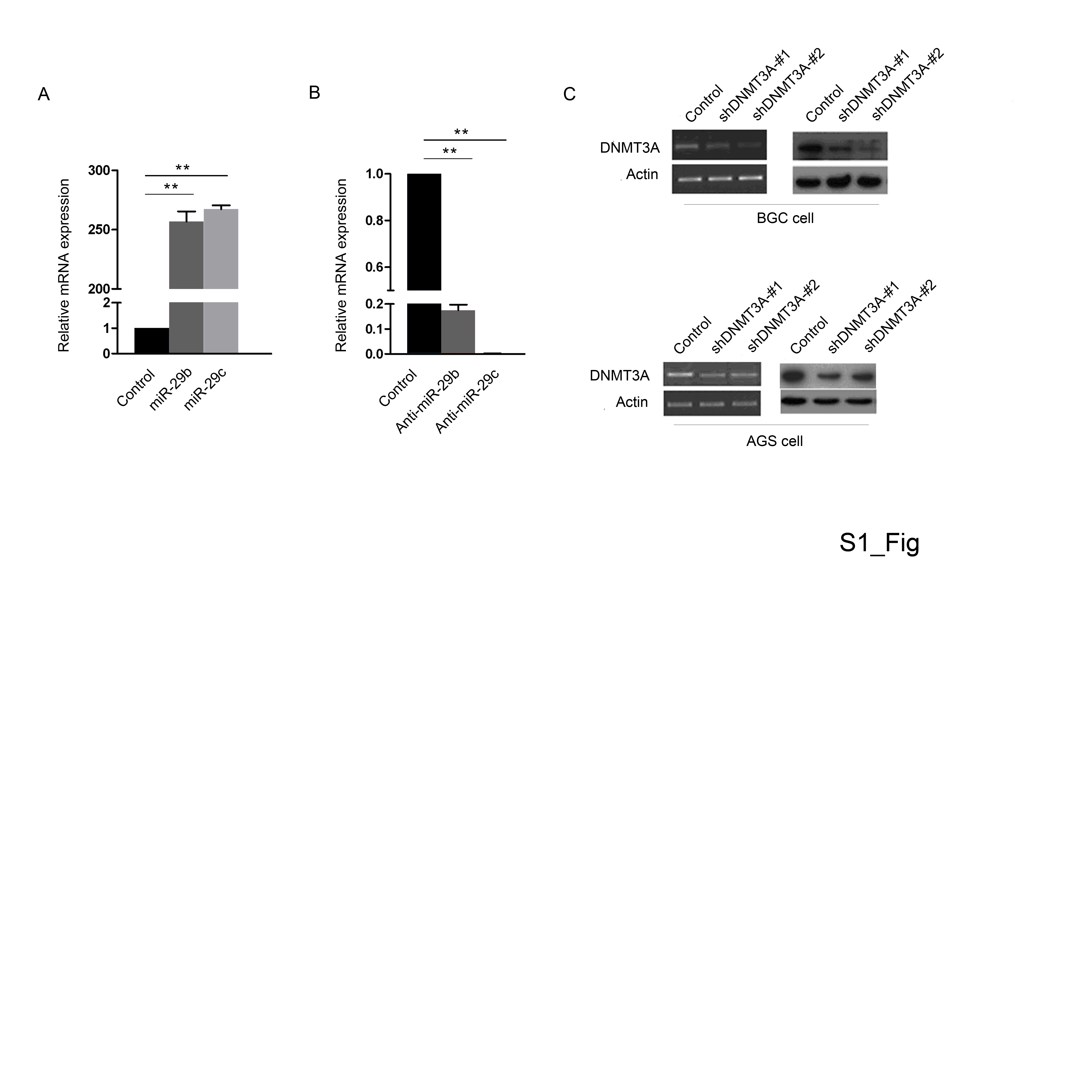

Supplement: S1 Fig — (A and B) qRT-PCR was performed to detect the relative expression of miR-29b/c in BGC-823 cells with mimics (A) or inhibitors (B) after 48-hour transfection. (C) RT-PCR and western blots were performed to detect the efficiency of DNMT3A knockdown in BGC and AGS cells. β-actin was used as a loading control. (TIF) [file pone.0123926.s001.tif]

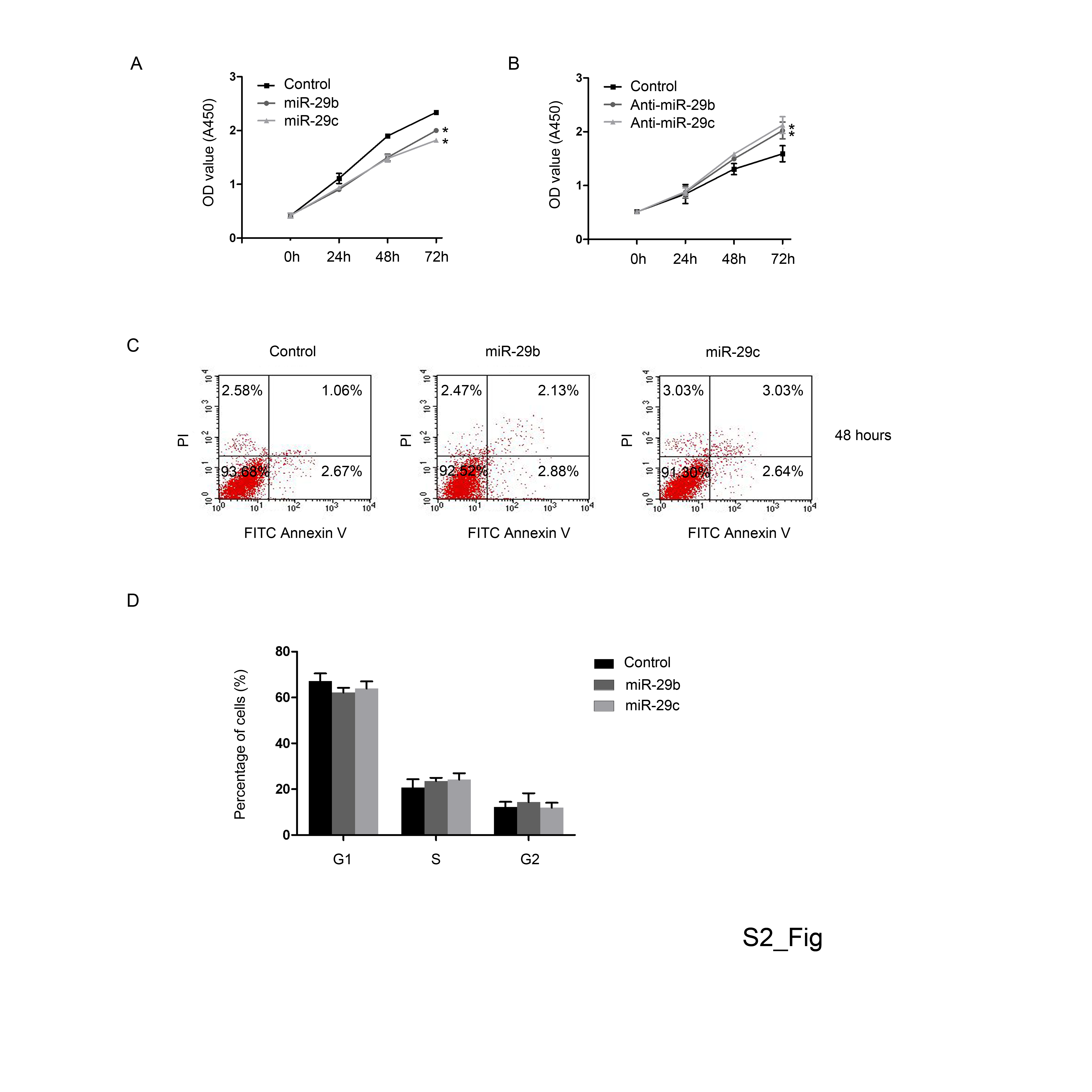

Supplement: S2 Fig — (A) The cell growth rates of miR-29b/c overexpression were detected by CCK-8 proliferation assay. miR-29b/c overexpression showed no remarkable difference at 48 hours compared to negative control cells (P>0.05). (B) The cell growth rates of miR-29b/c inhibition were detected by CCK-8 proliferation assay. The suppression of miR-29b/c showed no significant changes at 48 hours compared to negative control cells (P>0.05). (C) Apoptosis assay showing no dramatic induction of apoptosis by miR-29b/c overexpression at 48 hours compared to negative control cells. The biparametric histogram shows cell in early (bottom right quadrant) and the late apoptotic states (upper right quadrant). (D) The cell cycle assay was performed by flow cytometry on BGC-823 cells after miR-29b/c mimics or negative control mimics treatment for 48 hours. The percentages of miR-29b/c overexpression or control cells in the G1, S, and G2/M are shown in the bar chart as the mean±s.d. of three independent experiments. Compared with control cells, there was no significant effect on the cell cycle after miR-29b/c overexpression. (TIF) [file pone.0123926.s002.tif]
