# Supplementary material for: Deregulation between miR-29b/c and DNMT3A Is Associated with Epigenetic Silencing of the CDH1 Gene, Affecting Cell Migration and Invasion in Gastric Cancer
Source: PLoS One. 2015 Apr 15;10(4):e0123926. doi: 10.1371/journal.pone.0123926 (PMC4398372; doi:10.1371/journal.pone.0123926)
Supplement: S1 Method — (DOC) [file pone.0123926.s003.doc]

**S1 Method. Cell growth and apoptosis assay.** The cell counting kit-8 (CCK-8) (Dojindo Laboratories, Kumamoto, Japan) was used to measure the cellular growth of BGC-823 cells after treatment with miR-29b/c mimics/inhibitors and negative controls. Proliferation rates were determined at 24, 48, 72 hours post-transfection. The 450 nm absor­bance was measured to determine the cell viability. All of the experiments were independently repeated at least three times. Apoptosis was detected by PI and Annexin V-FITC staining. The BGC-823 cells were seeded at 3x10^5^ cells/well in 6-well plates and incubated for 48 hours. Trypsinized cells were washed three times with PBS. The cells were then conjugated with Annexin V-FITC using the PI/Annexin V-FITC kit (Biouniquer, USA), according to the manufacturer's protocol, and analyzed by flow cytometry (Olympus, Japan).
