# Supplementary material for: Deregulation between miR-29b/c and DNMT3A Is Associated with Epigenetic Silencing of the CDH1 Gene, Affecting Cell Migration and Invasion in Gastric Cancer
Source: PLoS One. 2015 Apr 15;10(4):e0123926. doi: 10.1371/journal.pone.0123926 (PMC4398372; doi:10.1371/journal.pone.0123926)
Supplement: S2 Method — (DOC) [file pone.0123926.s004.doc]

**S2 Method. Cell cycle assay.** Cell cycle analysis was performed on BGC-823 cells 48 hours after transfection with miR-29b/c mimics or negative control mimics. Approximately 1×10^6^ cells were trypsinized, washed twice with phosphate-buffered saline containing 1% fetal bovine serum and incubated in phosphate-buffered saline containing 0.02% TritonX-100, 0.1 mg/ml RNase (Sigma-Aldrich) and 10 μg/ml propidium iodide (Sigma-Aldrich) for 30 min at 37°C. The cell cycle distribution was examined by flow cytometry using a FACScan flow cytometer (Becton Dickinson & Co., San Jose, CA, USA). The relative number of cells in each phase of the cell cycle was analyzed using the Modfit program (Verity Software House Inc., Topsham, ME, USA).
