# Supplementary material for: Deregulation between miR-29b/c and DNMT3A Is Associated with Epigenetic Silencing of the CDH1 Gene, Affecting Cell Migration and Invasion in Gastric Cancer
Source: PLoS One. 2015 Apr 15;10(4):e0123926. doi: 10.1371/journal.pone.0123926 (PMC4398372; doi:10.1371/journal.pone.0123926)
Supplement: S1 Table — (DOC) [file pone.0123926.s005.doc]

| **S1­ Table Clinical features of patients with gastric cancer** | | | |
| --- | --- | --- | --- |
| **Sample number** | **Age** | **Gender** | **TNM staging** |
| 1 | 54 | female | Ⅰ |
| 2 | 73 | Female | Ⅲ |
| 3 | 41 | Male | Ⅱ |
| 4 | 74 | Female | Ⅰ |
| 5 | 62 | Male | Ⅱ |
| 6 | 78 | Male | Ⅰ |
| 7 | 55 | Male | Ⅲ |
| 8 | 53 | Male | Ⅲ |
| 9 | 62 | Male | Ⅱ |
| 10 | 72 | Male | Ⅲ |
| 11 | 65 | Male | Ⅲ |
| 12 | 73 | Male | Ⅱ |
| 13 | 73 | Male | Ⅱ |
| 14 | 75 | Male | Ⅲ |
| 15 | 62 | Female | Ⅱ |
| 16 | 43 | Male | Ⅲ |
| 17 | 45 | Male | Ⅱ |
| 18 | 74 | Male | Ⅱ |
| 19 | 62 | Male | Ⅱ |
| 20 | 72 | Female | Ⅲ |
| 21 | 72 | Male | Ⅱ |
| 22 | 60 | Female | Ⅰ |
| 23 | 91 | Female | Ⅳ |
| 24 | 44 | Male | Ⅰ |
| 25 | 46 | Male | Ⅳ |
| 26 | 70 | Male | Ⅱ |
| 27 | 77 | Female | Ⅲ |
| 28 | 64 | Male | Ⅲ |
| 29 | 70 | Female | Ⅰ |
| 30 | 34 | Male | Ⅲ |
| 31 | 71 | Male | Ⅲ |
| 32 | 72 | Male | No information |
| 33 | 75 | Female | Ⅱ |
| 34 | 74 | Male | Ⅱ |
| 35 | 60 | Female | Ⅱ |
| 36 | 62 | Male | Ⅱ |
| 37 | 73 | Male | Ⅱ |
| 38 | 72 | Female | No information |
| 39 | 73 | Male | No information |
| 40 | 74 | Male | No information |
| 41 | 57 | Female | No information |
| S1 Table (continued) | | | |
| **Sample number** | **Age** | **Gender** | **TNM staging** |
| 42 | 64 | Male | No information |
| 43 | 74 | Male | No information |
| 44 | 61 | Male | No information |
| 45 | 63 | Male | No information |
| 46 | 70 | Male | No information |
| 47 | 79 | Female | No information |
| 48 | 69 | Female | No information |
| 49 | 74 | Male | No information |
| 50 | 65 | Male | No information |
