# Supplementary material for: Deregulation between miR-29b/c and DNMT3A Is Associated with Epigenetic Silencing of the CDH1 Gene, Affecting Cell Migration and Invasion in Gastric Cancer
Source: PLoS One. 2015 Apr 15;10(4):e0123926. doi: 10.1371/journal.pone.0123926 (PMC4398372; doi:10.1371/journal.pone.0123926)
Supplement: S2 Table — (DOC) [file pone.0123926.s006.doc]

| **miRNA mimics/inhibitor sequences** | |
| --- | --- |
| miR-29b mimics | F:5′-UAGCACCAUCUGAAAUCGGUUA-3′ |
|  | R:5′-ACCGAUUUCAGAUGGUGCUAUU-3′ |
| miR-29c mimics | F:5′-UAGCACCAUUUGAAAUCGGUUA-3′ |
|  | R:5′-ACCGAUUUCAAAUGGUGCUAUU-3′ |
| mimics control | F:5′-UUCUCCGAACGUGUCACGUTT-3′ |
|  | R:5′-ACGUGACACGUUCGGAGAATT-3′ |
| miR-29b inhibitor | 5′-AACACUGAUUUCAAAUGGUGCUA-3 |
| miR-29c inhibitor | 5′-UAACCGAUUUCAAAUGGUGCUA-3′ |
| inhibitor control | 5′-CAGUACUUUUGUGUAGUACAA-3′ |
| **Primer in this study** |  |
| DNMT3A 3'-UTR | F:5′-ATTGCTAGCCGAAAAGGGTTGGACATCAT-3′ |
|  | R:5′-TTAGTCGACGCCGAGGGAGTCTCCTTTTA-3′ |
| DNMT3A | F: 5′- TATTGATGAGCGCACAAGAGAGC-3′ |
|  | R: 5′-GGGTGTTCCAGGGTAACATTGAG -3 |
| β-actin | F: 5′-AAAGACCTGTACGCCAACAC-3′ |
|  | R: 5′-GTCATACTCCTGCTTGCTGAT-3′ |
| miR-29b/c for BGS | F:5′-TAGTAGTGGTTGTTTGTTTTTTTGA-3′ |
|  | R:5′-CCACTCTACTAAAAACTCCATCTCC-3′ |

**S2 Table. miRNA mimic and inhibitor sequences and primers used in this study.**
